# Koumine inhibits osteoclastogenesis and prevents ovariectomy-induced bone loss via suppression of the MAPK signaling pathways

**DOI:** 10.1038/s41598-026-57325-4

**Published:** 2026-06-13

**Authors:** Xiang Zhang, Yang Wu, Kai-qi Jiang, Bang-sheng Cui, Jiong-Ming You

**Affiliations:** 1 Orthopedic Center, ShunDe Hospital of Guangzhou University of Chinese Medicine, Foshan, 528300 China; 2 Department of Gastroenterology, Guangdong Hospital of Integrated Traditional Chinese and Western Medicine, Foshan, 528253 China; 3 Nanchang Medical College, Nanchang, 330036 China; 4 Department of Orthopaedics, Wenzhou Hospital of Integrated Traditional Chinese and Western Medicine, Wenzhou, 325001 China

**Keywords:** Koumine, Osteoclastogenesis, Osteoporosis, MAPK signaling, Bone resorption, Cell biology, Diseases, Drug discovery, Medical research

## Abstract

Excessive osteoclast formation drives osteolytic bone diseases such as osteoporosis. Koumine (KM), an alkaloid derived from Gelsemium elegans, exhibits various bioactivities; however, its role in bone homeostasis remains unknown. This study investigated the effects of KM on RANKL-induced osteoclastogenesis in bone marrow-derived macrophages (BMMs). Cell viability, differentiation (TRAcP staining), and function (F-actin ring formation, bone resorption pit assay) were assessed. The underlying mechanisms were explored using Western blot and qPCR. The in vivo efficacy of KM was evaluated in an ovariectomized (OVX) mouse model using micro-CT and histological analyses. KM dose-dependently inhibited osteoclast formation and bone resorption without cytotoxicity. It suppressed RANKL-induced activation of the mitogen-activated protein kinase (MAPK) pathway, downregulating c-Fos, NFATc1, TRAP, and CTSK. In contrast, KM had no significant effect on RANKL-induced NF-κB activation. KM did not impair osteoblast differentiation or mineralization. In vivo, KM treatment prevented OVX-induced bone loss, improved trabecular microarchitecture, and reduced osteoclast numbers. KM suppresses osteoclastogenesis and protects against bone loss, an effect associated with inhibition of MAPK signaling, highlighting its potential as a novel therapeutic for osteolytic diseases.

**Supplementary Information:**

The online version contains supplementary material available at https://doi.org/10.1038/s41598-026-57325-4.

## Introduction

Osteoclasts are the principal cells responsible for bone resorption, and their excessive activation is a hallmark of osteolytic diseases such as postmenopausal osteoporosis and tumor-associated bone metastasis^1–4^. This type of bone metastasis is mainly divided into two categories: osteolytic and osteogenic. When osteolytic tumors, such as breast cancer and colon cancer, metastasize to the bone, they cause bone destruction^5^. This can lead to complications such as pathological fractures, hypercalcemia, and pain. In severe cases, which can lead to patient death^6,7^. Bone homeostasis is a dynamic equilibrium process between bone formation and bone resorption. Any enhancement or weakening of either side can disrupt bone homeostasis, leading to bone destruction. Breast cancer is the most common cancer type in women, and bone is one of the most frequent sites of distant metastasis in breast cancer patients^8^. The incidence of bone metastasis in patients with advanced breast cancer is 65−75%, and 27−50% of patients present with bone metastasis as their initial symptom^9^. The pathogenesis of bone destruction in breast cancer metastasis involves tumor cell migration and proliferation, along with excessive osteoclast activation and differentiation. Indeed, aberrant osteoclast formation and function are recognized as key drivers of osteolysis in this context^10–12^. Under normal physiological conditions, bone homeostasis is tightly regulated by the coupled activity of osteoblasts and osteoclasts^13,14^. Pathological over-activation of osteoclasts disrupts this balance, leading to osteolytic diseases^15–17^. Consequently, inhibiting osteoclast formation is a major therapeutic strategy for osteolytic bone metastases.

Osteoclasts are multinucleated giant cells derived from hematopoietic precursor cells of the monocyte/macrophage lineage^18^. Macrophage colony-stimulating factor (M-CSF)and receptor activator of nuclear factor-κB (NF-κB)ligand (RANKL)are key cytokines involved in osteoclast formation and differentiation^19^. M-CSF promotes the proliferation and survival of osteoclast precursors and upregulates the expression of RANK^20,21^. RANKL binds to its receptor RANK and sequentially recruits tumor necrosis factor receptor-associated factor 6 (TRAF6)in osteoclast precursors. TRAF6 activates downstream signaling pathways, including NF-κB and mitogen-activated protein kinases (MAPKs)^22,23^. This leads to the activation and accumulation of two key transcription factors involved in osteoclast differentiation, namely c-Fos and nuclear factor of activated T cells c1 (NFATc1)^24–26^. These factors induce the expression of osteoclast-specific genes, including tartrate-resistant acid phosphatase (TRAP), dendritic cell-specific transmembrane protein (DC-STAMP), cathepsin K (CTSK), and calcitonin receptor (CTR), thereby forming mature osteoclasts^27^.

Koumine (KM), an indole alkaloid isolated from Gelsemium elegans, has been reported to exhibit diverse pharmacological activities including antitumor, anti-inflammatory, and immunomodulatory activities^28–32,33,34^. Recently, our team demonstrated that KM inhibits RANKL-induced NF-κB activation via ubiquitination, thereby preventing bone loss in ovariectomized and aging mice^47^. However, that study focused primarily on ubiquitination and did not address whether KM simultaneously modulates the MAPK pathway—a parallel signaling cascade critically involved in osteoclastogenesis. Furthermore, the specificity of KM for osteoclasts versus osteoblasts, and its direct impact on osteoclastic bone resorption, were not investigated. Given the pivotal role of the RANKL-induced MAPK and NF-κB pathways in osteoclastogenesis, we hypothesized that KM might protect against bone loss by targeting these pathways. Therefore, in the present study, we aimed to build upon these foundational findings by comprehensively evaluating the effects of KM on MAPK signaling and assessing whether NF-κB activation is also affected. We also assessed its functional consequences on bone resorption and determined its selectivity for osteoclasts over osteoblasts using an ovariectomy-induced osteoporosis model in vivo.

## Materials and methods

### Materials and reagents

The alpha modification of Eagle’s medium (α-MEM), Dulbecco’s Modified Eagle Medium (DMEM), Penicillin/streptomycin (PS)and fetal bovine serum (FBS)were purchased from HyClone company (GE Healthcare Life Sciences, Chicago, IL, USA). KM with a purity of 99.5% was obtained from Shanghai yuanye Bio-Technology Co., Ltd and first dissolved in 100% dimethyl sulfoxide (DMSO)as a stock solution. Then it was diluted by αMEM before cell culture to ensure that the final concentration of DMSO was less than 0.1% in the total culture medium. Antibodies specific for GAPDH (14C10), Phospho-IκBα (Ser32) (14D4), Phospho-NF-κB p65 (Ser536) (93H1), NF-κB p65 (C22B4), IκBα (44D4), NFATC1 were purchased from Cell Signaling Technology (CST, Danvers, MA, USA). RIPA cell lysis buffer, phenylmethylsulfonyl fluoride (PMSF), proteaseinhibitor and phosphatase inhibitor were from Beyotime Institute of Biotechnology (Jiangsu, China). Macrophage colony stimulating factor (M-CSF)and receptor activator of nuclear factor κB ligand (RANKL)were purchased from R&D Systems (Minneapolis, MN, USA).

### Cell culture

Primary bone marrow cells were isolated by flushing the femurs and tibiae of 6-week-old C57BL/6J mice. These cells were then cultured in complete α-MEM medium, which contained 10% FBS, 1% penicillin/streptomycin, and 100 ng/mL M-CSF to promote the proliferation of bone marrow-derived macrophages (BMMs). The cells were cultured at 37 °C in a humidified 5% CO₂ incubator for approximately 4 days. After washing with PBS, the adherent cells were harvested and used as bone marrow-derived macrophages (BMMs)for subsequent experiments.

### CCK-8 assay for cell viability

To evaluate cell viability, BMMs were plated in 96-well plates and treated with a range of KM concentrations for up to 96 h. Following the incubation, 10 µL of CCK-8 reagent was added to each well, and the plates were kept in the dark for 2 h. The resulting absorbance was quantified at 450 nm using a microplate reader. (BioTek Instruments, Winooski, VT, USA).

### Tartrate resistant acid phosphatase (TRAcP) staining

BMMs (8 × 10^3 cells/well)were seeded in a 96-well plate and cultured in an osteoclast differentiation medium containing 100 ng/mL M-CSF and 100 ng/mL RANKL, with or without various concentrations of KM (0, 10, 20, and 40 µM)for 5 d. Upon the appearance of mature osteoclasts, cultures were fixed in 4% paraformaldehyde and subsequently subjected to staining using a commercial TRAcP staining kit, following the manufacturer’s protocol. TRAcP-positive multinucleated cells (defined as osteoclasts with three or more nuclei)were then observed under light microscopy.

### Functional bone resorption assay

BMMs were seeded in 6-well plates containing osteoclast differentiation medium and cultured for 4–5 days. Once pre-osteoclasts had formed, they were harvested using 0.25% trypsin, gently detached, and collected by centrifugation at 1000×g for 5 min. The cells were then resuspended and equally reseeded onto 96-well hydroxyapatite-coated plates. After 10 h for adherence, the cells were cultured in stimulation medium with or without various concentrations of KM (0, 10, 20, and 40 µM). Four days later, the adherent cells were incubated with 5% sodium hypochlorite solution for 15 min and then washed three times with sterile water. After drying, the plates were examined under a light microscope, and the area of resorption pits was quantified as a percentage of the total surface area using ImageJ software.

### Quantitative real-time PCR (qPCR)

After being cultured with osteoclast differentiation medium in the presence or absence of KM for 5 d, osteoclasts were generated from BMMs. Total RNA was isolated from the cells using TRIzol reagent (Thermo Fisher Scientific)according to the manufacturer’s protocol. cDNA was generated from the RNA samples using PrimeScript RT Master Mix (Takara Bio). Real-time quantitative PCR (qPCR)was performed on a qTOWER Real-time PCR Thermal Cycler (Analytik Jena, Jena, Germany) using mixtures of SYBR Green PCR Master Mix (Thermo Fisher Scientific), cDNA, and forward and reverse primers. The PCR cycling parameters were as follows: 95℃ for 3 min, followed by 40 cycles of 95℃ for 10 s, 60℃ for 20 s, and 72℃ for 20 s, with a final extension step at 72℃ for 20 s. Table 1 lists the specific primer sequences. V-ATPase-a3 mRNA expression was detected using primers targeting its encoding gene, Atp6v0d2.

**Table 1:** Sequences of oligonucleotides used as primers.

Target gene	Forward	Reverse
*Gapdh*	5′-AGGTCGGTGTGAACGGATTTG−3′	5′-GGGGTCGTTGATGGCAACA−3′
*c-Fos*	5′-GCGAGCAACTGAGAAGAC−3′	5′-TTGAAACCCGAGAACATC−3′
*Nfatc1*	5′-CAA GCCCTGACCACCGATAG−3′	5′-GGCTGCCTTCCGTCTCATAGT−3′
*Ctsk*	5′-GGGAGAAAAACCTGAAGC−3′	5′-ATTCTGGGGACTCAGAGC−3′
*Trap*	5′-TCCTGGCTCAAAAAGCAGTT−3′	5′-ACATAGCCCACACCGTTCTC−3′
*Atp6v0d2*	5′-GTGAGACCTTGGAAGACCTGAA−3′	5′-GAGAAATGTGCTCAGGGGCT−3′

### Western blotting

BMMs were serum-starved for 1 h, pre-treated with or without 20 µM KM for 3 h, and then stimulated with 50 ng/mL RANKL for 5, 10, 20, 30, or 60 min. The concentration of 20 µM was selected for Western blotting to minimize potential non-specific cellular stress while allowing detection of signaling changes. Total protein was extracted from cells using RIPA lysis buffer supplemented with protease and phosphatase inhibitors. Protein samples (30 µg per lane)were then resolved by SDS-PAGE and electrophoretically transferred onto nitrocellulose membranes using the Trans-Blot Turbo Transfer System (Bio-Rad Laboratories, Hercules, CA, USA). The membranes were blocked with 5% skim milk for 1 h and then washed three times with TBST (Tris-buffered saline containing 0.1% Tween 20). The membranes were then incubated with specific primary antibodies (diluted 1:1,000)overnight at 4 °C, followed by incubation with the corresponding fluorescently labeled secondary antibodies (IRDye; diluted 1:1,000)for 1 h at room temperature. Protein bands were visualized using the Odyssey Imaging System (LI-COR Biosciences, Lincoln, NE, USA).

### Osteoblast differentiation and mineralization assay

Primary osteoblasts were isolated from calvariae of neonatal (1–3 day old) C57BL/6J mice. Briefly, calvariae were dissected, minced, and digested with 0.25% trypsin-EDTA for 10 min, followed by four sequential digestions with 0.1% collagenase type I for 15 min each. Cells from the last three digestions were pooled, cultured in α-MEM containing 10% FBS and 1% penicillin/streptomycin, and used at passage 2–3 for all experiments. For osteogenic differentiation, cells were seeded in 24-well plates at a density of 2 × 10⁴ cells/well. After reaching confluence, the culture medium was replaced with osteogenic induction medium consisting of α-MEM supplemented with 10% FBS, 10 mM β-glycerophosphate, 50 µg/mL ascorbic acid, and 10 nM dexamethasone, with or without KM (20 and 40 µM). The medium was changed every 3 days. Alkaline phosphatase (ALP) staining was performed after 7 days of induction. Cells were fixed with 4% paraformaldehyde for 15 min and stained using a BCIP/NBT ALP staining kit (Beyotime, China) according to the manufacturer’s instructions. ALP activity was quantified using p-nitrophenyl phosphate (pNPP) as a substrate; absorbance was measured at 405 nm. For mineralization assessment, cells were cultured in osteogenic induction medium for 21 days, then fixed with 4% paraformaldehyde and stained with 2% Alizarin Red S (pH 4.2) for 30 min. Stained nodules were visualized under light microscopy, and quantification was performed by destaining with 10% cetylpyridinium chloride for 1 h, followed by absorbance measurement at 562 nm. For gene expression analysis, total RNA was extracted after 7 days of induction, and qPCR was performed as described in Sect. 2.6. The primer sequences for osteogenic markers were: ALP forward 5’-GCTGATCATTCCCACGTTTT-3’, reverse 5’-CTGGGCCTGGTAGTTGTTGT-3’; Osteopontin forward 5’-GATGGCCGAGGTGATAGCTT-3’, reverse 5’-GGTCTCATCATTGGCTTCTC-3’; and GAPDH as an internal control (primers listed in Table 1).

### OVX-induced bone loss model

All animal experiments were approved by the Animal Ethics Committee of Guangzhou University of Chinese Medicine (Approval No. 20231018001). All methods were carried out in accordance with relevant guidelines and regulations, including the ARRIVE guidelines (https://arriveguidelines.org) for reporting animal research, and complied with the institutional ethical standards. Female C57BL/6J mice were purchased from Changzhou CAVENS and housed in the Animal Research Center at Guangzhou University of Chinese Medicine (Guangzhou, China). The housing conditions were maintained at a constant temperature of 22 ± 1 °C, relative humidity of 55 ± 5%, and a 12/12-h light/dark cycle. Bedding was replaced every 3 d, and all mice had free access to water and food throughout the experiment. After a 1-week acclimation period, 24 female 11-week-old C57BL/6J mice were randomly divided into four groups (n = 6): sham, OVX, OVX + 1 mg/kg KM, and OVX + 10 mg/kg KM. Mice were anesthetized by inhalation of 2–3% isoflurane in 100% oxygen at a flow rate of 0.5 L/min in an induction chamber. Anesthesia depth was confirmed by the absence of a toe pinch reflex. During surgery, anesthesia was maintained via a nose cone with 1.5–2% isoflurane. Under anesthesia with isoflurane gas, bilateral OVX was performed on all mice except those in the sham group. Mice in the sham group underwent the same surgical procedure, but the ovaries were exteriorized and not resected. Following a one-week post-operative recovery period, the OVX + KM treatment groups were administered KM via intraperitoneal injection at doses of 1 or 10 mg/kg every other day, continuing for a total duration of six weeks. Mice in the sham and OVX groups received intraperitoneal injections of an equivalent volume of normal saline as a vehicle control. After 6 weeks of treatment, all mice were euthanized by cervical dislocation under deep isoflurane anesthesia (5% isoflurane for 5 minutes), followed by confirmation of death via cardiac puncture and cessation of respiration. Tibias were immediately collected for subsequent analyses. The doses of KM (1 and 10 mg/kg) were selected based on a preliminary dose-ranging study (data not shown) and previous literature (You et al., 2024^47^), in which these doses effectively prevented bone loss without observable toxicity.”

### Micro-CT and histological analysis

The tibias were placed into a scanning tube and imaged using a Skyscan 1272 micro-CT scanner (Bruker micro-CT, Kontich, Belgium)with the following parameters: voltage, 50 kV; current, 450 µA; and isotropic resolution, 9 μm. The microstructural properties of the trabecular bone, including bone volume/total volume (BV/TV), trabecular number (Tb.N), trabecular spacing (Tb.Sp), and bone surface to total volume ratio (BS/TV), were analyzed. Following micro-CT scanning, the tibias were decalcified in EDTA decalcifying solution for approximately 4 weeks, dehydrated through a graded ethanol series, cleared in xylene, and carefully embedded in paraffin. Section (5 μm thick)were cut and subjected to hematoxylin-eosin (H&E)staining to observe the bone microstructure and tartrate-resistant acid phosphatase (TRAP)staining to visualize osteoclasts.

### Statistical analysis

All data are representative of at least three independent experiments performed in triplicate, unless otherwise indicated. Data are presented as the mean ± standard deviation (SD). Statistical analysis was performed using one-way ANOVA followed by Tukey’s multiple comparisons test to determine the significance of differences between groups. Differences with a p value < 0.05 were considered statistically significant.

## Results

### KM inhibits RANKL-induced osteoclastogenesis and function in vitro

**Fig. 1:**
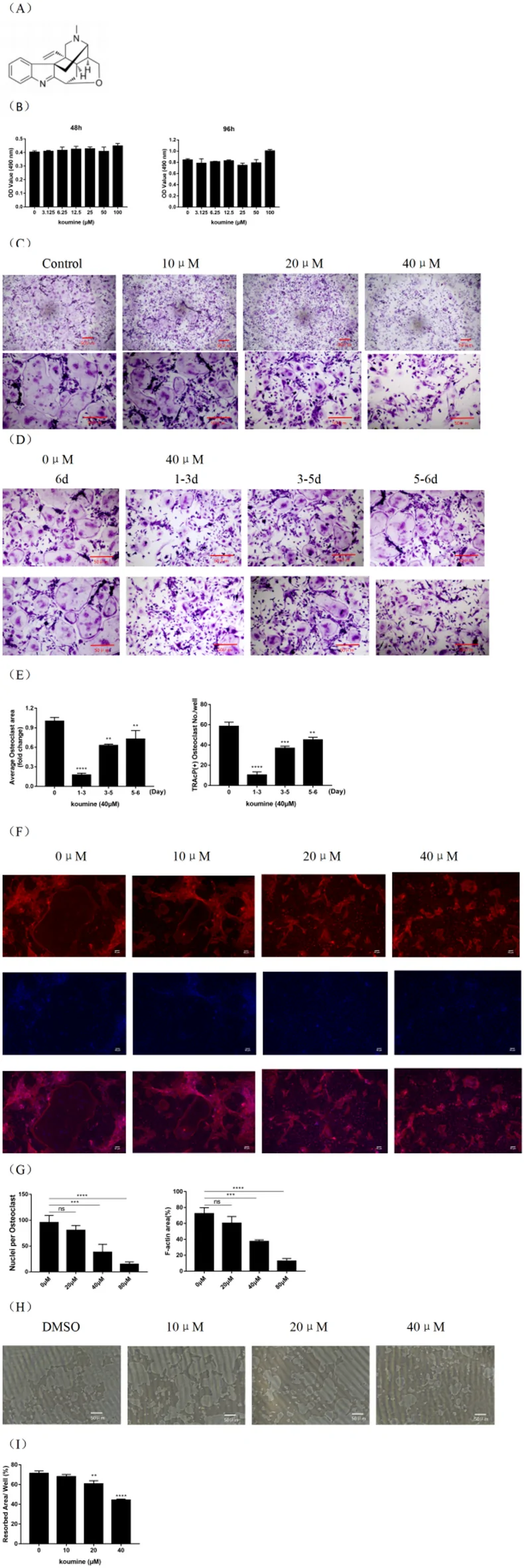
KM inhibits RANKL-induced osteoclastogenesis and function in vitro. (**A**) Structural formula of KM. (**B**) Viability of bone marrow-derived macrophages (BMMs)treated with the indicated concentrations of KM for 48 and 96 h, as determined by CCK-8 assay. (**C**) Representative images (upper) and quantification (lower) of TRAcP staining of BMMs treated with RANKL and the indicated concentrations of KM for 5 days. Osteoclasts are defined as TRAcP-positive cells with three or more nuclei. (**D**) Time-dependent inhibitory effect of KM (40 µM) on osteoclast formation. KM was added at different time windows during the 6-day RANKL induction period. Representative images (left) and quantification (right) of TRAcP-stained osteoclasts are shown. (**F**) Representative fluorescence images of F-actin (red, stained by phalloidin)and nuclei (blue, stained by DAPI)in osteoclasts treated with RANKL and the indicated concentrations of KM. The number of actin rings per well (G) was quantified. (H)Representative images of resorption pits on hydroxyapatite-coated plates by osteoclasts treated with RANKL and the indicated concentrations of KM. The resorbed area (I)was quantified and is presented as a percentage of the total area. All quantitative data are presented as mean ± SD (*n* = 5). *p < 0.05, **p < 0.01, ***p < 0.001, ****p < 0.0001.

The concentrations of KM (10, 20, and 40 µM) were selected based on a preliminary dose-finding experiment (data not shown) and the CCK-8 assay results (Fig. 1B), which demonstrated no cytotoxicity up to 100 µM. Prior to evaluating the effect of KM (Fig. 1A)on osteoclastogenesis, we first confirmed its lack of cytotoxicity on bone marrow-derived macrophages (BMMs)using a CCK-8 assay. Treatment with KM for up to 96 h did not impair BMM viability at concentrations as high as 100 µM (Fig. 1B), indicating that its subsequent effects are not attributable to cellular toxicity. We then investigated the impact of KM on RANKL-induced osteoclast differentiation through both concentration-dependent and time-dependent assays. KM potently inhibited osteoclast formation in a concentration-dependent manner, with 40 µM exhibiting a particularly strong effect (Fig. 1C). This inhibitory effect was most pronounced when KM was administered during the early stages of differentiation (days 1–3) (Fig. 1D, E). Furthermore, functional assays revealed that KM disrupted the formation of actin rings, a prerequisite for bone resorption, and significantly reduced the number of nuclei per osteoclast (Fig. 1F, G). Consistent with this, KM dose-dependently inhibited osteoclastic bone resorption, as evidenced by a reduction in pit formation on hydroxyapatite-coated plates (Fig. 1H, I). As KM was administered during the differentiation phase (from pre-osteoclasts onward), this reduction likely reflects both impaired osteoclast formation and a potential direct suppression of the resorptive capacity of the osteoclasts that did form. Collectively, these results demonstrate that KM effectively suppresses both the formation and the bone-resorptive function of osteoclasts in vitro.

### KM suppresses RANKL-induced activation of ERK and p38, but not JNK or NF-κB

**Fig. 2:**
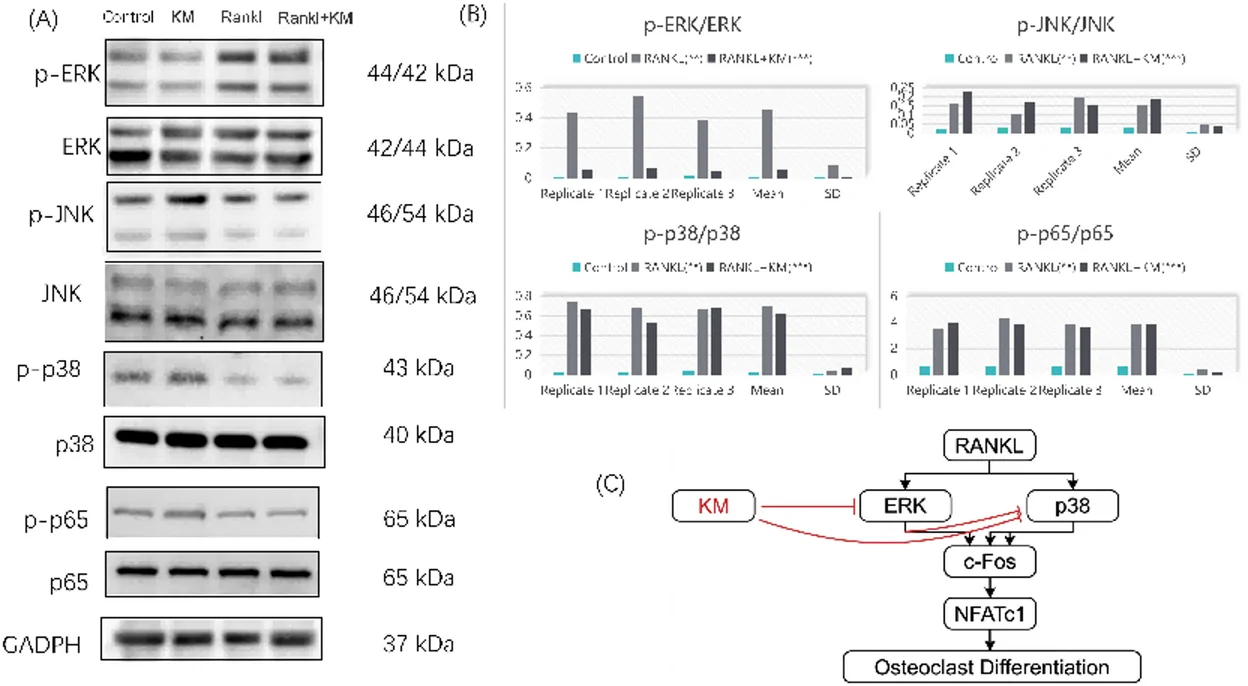
KM suppresses RANKL-induced activation of ERK and p38 MAPK signaling, but not JNK or NF-κB p65, in BMMs. (**A**) Bone marrow-derived macrophages (BMMs) were pretreated with or without 20 µM KM for 3 h, then stimulated with RANKL (50 ng/mL) for 15 min. Cell lysates were subjected to Western blot analysis using antibodies against p-ERK, ERK, p-JNK, JNK, p-p38, p38, p-p65, p65, and GAPDH. Representative blots from three independent experiments are shown. Molecular weights are indicated on the right. (**B**) Densitometric quantification of phosphorylated protein levels. The ratios of phosphorylated to total protein were quantified and normalized to the Control group. Data are presented as mean ± SD (*n* = 3 independent experiments). *p < 0.05, **p < 0.01, ***p < 0.001 vs. RANKL group; ns, not significant. (**C**) Schematic diagram of KM’s inhibitory mechanism. Based on the Western blot results shown in A–B, KM suppresses RANKL-induced osteoclastogenesis by inhibiting the phosphorylation of ERK and p38, leading to downregulation of c-Fos and NFATc1. KM had no statistically significant effect on the phosphorylation of JNK or NF-κB p65 under the conditions tested. The effect of KM on upstream signaling molecules such as TRAF6 was not investigated in this study and remains to be determined.**Full-length, uncropped blots are provided in Supplementary Fig. S1.**.

To elucidate the molecular mechanisms by which KM inhibits osteoclast differentiation, we examined its effect on RANKL-activated signaling pathways. Bone marrow-derived macrophages (BMMs) were pretreated with or without 20 µM KM for 3 h, followed by stimulation with RANKL (50 ng/mL) for 15 min. Western blot analysis revealed that RANKL stimulation strongly induced the phosphorylation of ERK, JNK, p38, and NF-κB p65 (Fig. 2A). KM pretreatment significantly attenuated the RANKL-induced phosphorylation of ERK and p38. However, KM had no significant effect on RANKL-induced phosphorylation of NF-κB p65 (Fig. 2A, B). JNK phosphorylation was not significantly altered by KM treatment (Fig. 2A).

Densitometric quantification of three independent experiments confirmed these observations (Fig. 2B). KM reduced p-ERK levels by approximately 88% (*p* < 0.001) and p-p38 levels by 10% (*p* < 0.05) compared to the RANKL-alone group. In contrast, the reduction in p-p65 levels was only 2%, which was not statistically significant. JNK phosphorylation showed a slight, non-significant increase of 20% in the KM-treated group (Fig. 2B).

These results demonstrate that KM selectively suppresses RANKL-induced activation of ERK and p38, while having no significant effect on JNK or NF-κB p65 phosphorylation under the conditions tested. It should be noted, however, that the lack of effect on p65 phosphorylation does not entirely rule out NF-κB involvement. KM may still influence NF-κB activity through alternative, phosphorylation-independent mechanisms such as modulating its nuclear translocation or DNA-binding affinity as suggested by our previous findings on ubiquitination (You et al.^47^).

Based on these findings, we propose a working model for KM’s inhibitory mechanism (Fig. 2C): KM inhibits RANKL-induced osteoclastogenesis by suppressing the phosphorylation of ERK and p38, leading to downregulation of NFATc1 and subsequent inhibition of osteoclast-specific gene expression and bone resorption.

### KM does not impair the viability, differentiation, or mineralization of osteoblasts

**Fig. 3:**
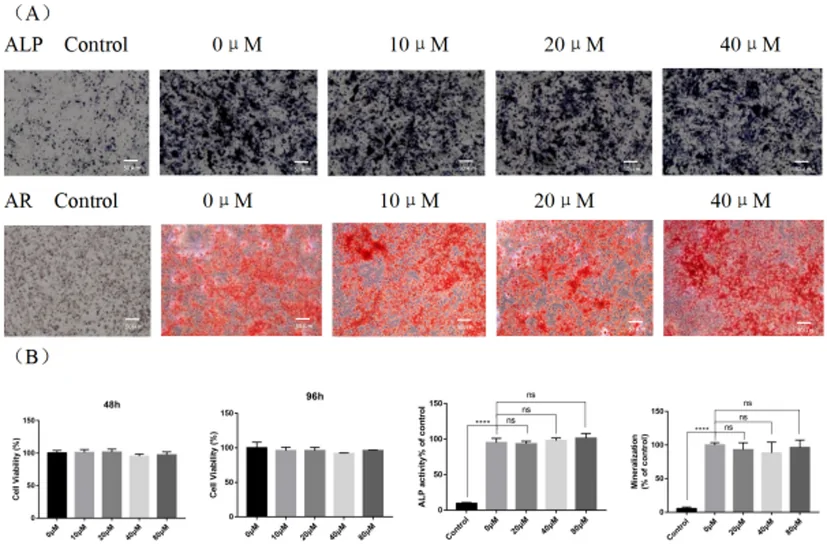
KM does not impair the viability, differentiation, or mineralization of osteoblasts. (**A**) Representative images of alkaline phosphatase (ALP) staining (after 7 days of osteogenic induction) and Alizarin Red S (ARS) staining (after 21 days of osteogenic induction) of bone marrow mesenchymal stem cells (BMSCs)treated with the indicated concentrations of KM. (**B**) Quantitative analysis of (A): ALP activity (upper left), mineralized nodule area (upper right), and the relative mRNA expression levels of the osteogenic markers ALP (lower left) and Osteopontin (lower right) in BMSCs. Data are presented as mean ± SD (*n* = 5). NS, not significant; ****p < 0.0001 versus the control group.

To ensure that the inhibitory effect of KM was specific to osteoclasts, we evaluated its influence on osteoblast viability and differentiation. First, CCK-8 assays^35^ confirmed that KM was non-toxic to calvaria-derived osteoblast precursors at concentrations up to 80 µM (Fig. 3B). We then examined its effects on osteogenic differentiation. After 7 days of induction, KM (20 and 40 µM) did not alter ALP activity or the expression of osteogenic genes like ALP and Osteopontin (Fig. 3A, B). Finally, after a 21-day induction period, KM treatment showed no inhibitory effect on matrix mineralization, as visualized and quantified by Alizarin Red S staining (Fig. 3A, B). These data collectively indicate that KM does not compromise osteoblast function.

### KM protects against OVX-induced bone loss in vivo

**Fig. 4:**
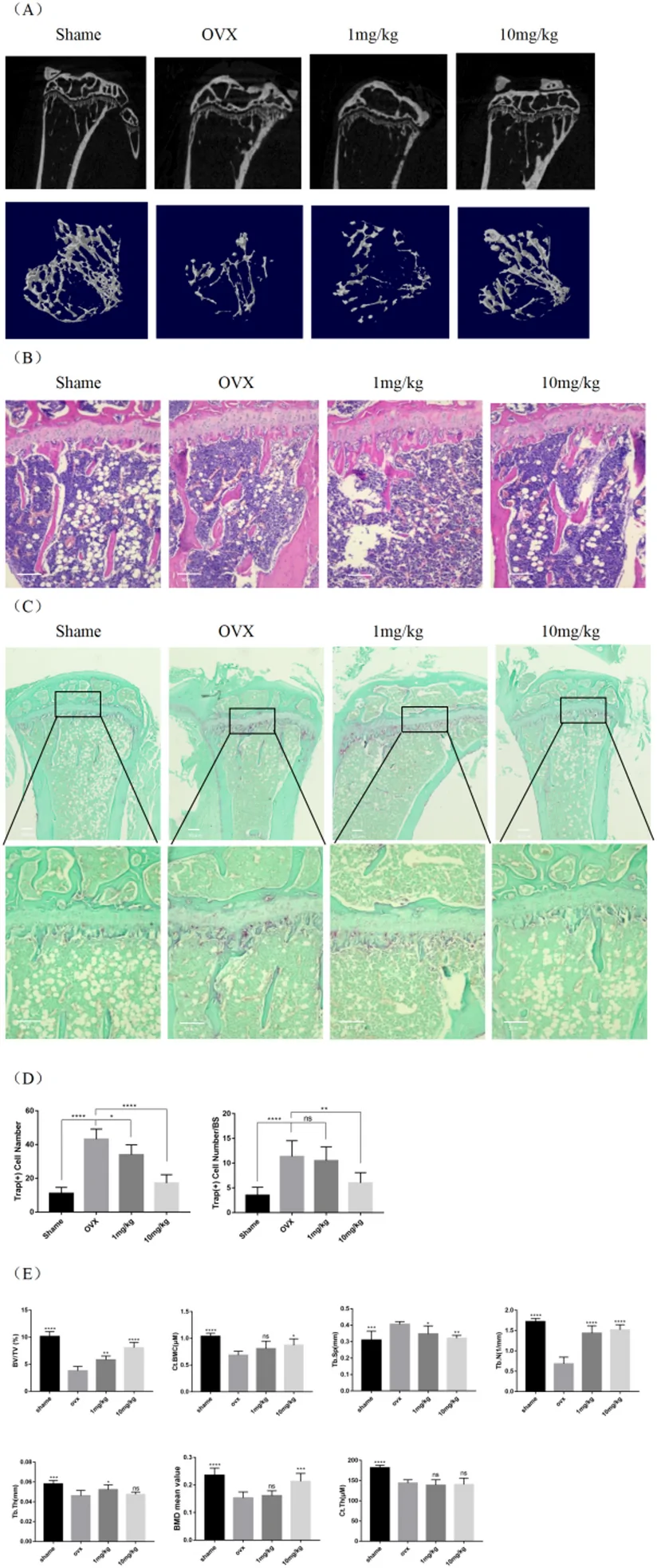
KM protects against OVX-induced bone loss in vivo. (**A**) Representative three-dimensional micro-CT reconstruction images of tibiae from the indicated groups. (**B**) Representative hematoxylin and eosin (H&E) staining of tibial sections, showing the trabecular bone microstructure. (**C**) Representative images of tartrate-resistant acid phosphatase (TRAcP) staining of tibial sections at low (×40) and high (×200) magnification. (**D**) Quantification of the number of TRAcP-positive osteoclasts per bone surface (N.Oc/BS) from (**C**). (**E**) Quantitative analysis of bone microarchitecture parameters from micro-CT: bone mineral density (BMD), bone volume/tissue volume (BV/TV), cortical bone mineral content (Ct.BMC), trabecular thickness (Tb.Th), trabecular number (Tb.N), and trabecular separation (Tb.Sp). Data are presented as mean ± SD (*n* = 6). *p < 0.05, **p < 0.01, ***p < 0.001 versus the OVX group.

To evaluate the therapeutic potential of KM in vivo, we employed an OVX -induced bone loss model in mice^36–38^. Three-dimensional micro-CT reconstruction and quantitative analysis revealed that OVX induction led to a significant deterioration of trabecular bone microstructure (Fig. 4A), characterized by decreased bone mineral density (BMD), bone volume fraction (BV/TV), trabecular number (Tb.N), and increased trabecular separation (Tb.Sp)compared to the Sham group. KM treatment (1 and 10 mg/kg)for 6 weeks effectively prevented these changes in a dose-dependent manner, with the high-dose group showing superior efficacy (Fig. 4A, E). Histological analysis corroborated these findings; H&E staining showed that KM ameliorated OVX-induced trabecular bone loss (Fig. 4B), and TRAP staining demonstrated that KM significantly reduced the increased number of osteoclasts observed in the OVX group (Fig. 4C, D). The in vivo data robustly demonstrate that KM treatment effectively prevents bone loss in OVX mice, and the concomitant reduction in osteoclast numbers confirms that this protective effect is mediated through the inhibition of osteoclast activity.

## Discussion

An increasingly aging population has led to a rising global incidence of skeletal disorders, including osteoporosis and bone metastases^8^. Breast cancer, a leading cause of cancer-related mortality in women, frequently metastasizes to bone, severely impacting physical and mental health^8,9^. Once breast cancer metastasizes to the bone, it disrupts normal bone remodeling, leading to pathological bone loss (osteolysis)^5,6^. It is currently believed that osteolytic bone metastasis is primarily driven by osteoclasts^16^. Breast cancer cells secrete factors that induce osteoclast formation, while the bone resorption process mediated by osteoclasts releases growth factors from the bone matrix, further promoting tumor growth in a vicious cycle^39^. Because the pathological process of breast cancer-induced bone destruction is similar to that of osteoporosis, drugs targeting osteoclasts are cornerstone therapies for both conditions^40^.

This integrative study, spanning molecular analyses, cellular assays, and in vivo validation, establishes KM as a novel and specific therapeutic candidate capable of inhibiting osteoclast formation and function. This study demonstrates for the first time that KM exerts its anti-osteoclastogenic effects not by cellular toxicity, but through suppression of the MAPK signaling pathway activated by RANKL. Although NF-κB is a critical downstream mediator of RANKL, our data showed that KM did not significantly inhibit RANKL-induced p65 phosphorylation, suggesting that the anti-osteoclastogenic effect of KM is primarily mediated through MAPK rather than NF-κB. Furthermore, its efficacy in preventing OVX-induced bone loss, coupled with its lack of adverse effects on osteoblast function, positions KM as a promising candidate for treating osteolytic diseases^15,40^.

The in vitro data offer a clear mechanistic insight into how KM disrupts the RANKL signaling cascade. M-CSF and RANKL are the two key cytokines for osteoclast formation and differentiation^19,41^, which regulate signaling cascades leading to the activation of critical transcription factors like c-Fos and NFATc1^24–26^. We found that KM pretreatment suppressed the RANKL-induced phosphorylation of ERK and p38, while having no significant effect on JNK or NF-κB p65 phosphorylation (Fig. 2). The NF-κB and MAPK pathways are among the most pivotal signaling cascades affecting osteoclast differentiation^23,42^. The phosphorylation of MAPKs (ERK, JNK, and p38)is known to contribute to RANKL-stimulated osteoclastogenesis and bone resorption, and specific inhibitors of these pathways can block the process^43,44^. Our findings that KM inhibits the phosphorylation of ERK and p38 are consistent with this paradigm, while the lack of effect on JNK suggests a selective inhibitory profile. An interesting observation was a slight (≈ 20%), albeit statistically non-significant, increase in JNK phosphorylation upon KM treatment (Figure. 2B). While not definitive, this trend could reflect compensatory cross-talk within the MAPK network, where inhibition of ERK/p38 leads to rebound JNK activation. Importantly, this modest elevation did not counteract the overall anti-osteoclastogenic effect of KM (Figure. 1). This early dual inhibition is crucial, as it likely disrupts the subsequent activation of the master transcription factor NFATc1^24,45^, ultimately leading to the downregulation of osteoclast-specific genes (e.g., TRAP, CTSK)^27^. This molecular-level inhibition directly translates to the observed cellular phenotypes: impaired osteoclast differentiation (Fig. 1C-E), disrupted actin ring formation (Fig. 1F, G), and abolished bone resorptive function (Fig. 1H, I). In a recent study from our group, (You et al., 2024^47^) demonstrated that KM inhibits NF-κB activation by targeting ubiquitination in osteoclasts. However, in the present study, we did not observe a significant reduction in RANKL-induced p65 phosphorylation upon KM treatment (Figure. 2). This apparent discrepancy may be attributed to differences in experimental conditions, such as KM concentration, stimulation time, or cell passage number. More importantly, this discrepancy highlights a potentially more nuanced mechanism: KM may not block the immediate phosphorylation event (p65 Ser536), but could interfere with upstream ubiquitination-dependent signaling steps (e.g. TRAF6 or NEMO ubiquitination), thereby affecting later events such as p65 nuclear translocation or transcriptional activity without necessarily altering its phosphorylation status at the residue examined. While the present study focused on phosphorylation, the possibility that KM influences NF-κB nuclear localization or DNA-binding affinity warrants further investigation. Future studies examining p65 nuclear localization by immunofluorescence and NF-κB luciferase reporter activity in KM-treated cells would help resolve this issue.

Importantly, the present study extends these observations in several key aspects. First, we show that KM concurrently suppresses ERK and p38 phosphorylation, while having no significant effect on JNK (Fig. 2)-a finding not addressed in the previous report. Second, we provide direct evidence that KM abrogates osteoclastic bone resorption via pit formation assays, linking pathway inhibition to functional outcomes. Third, we demonstrate that KM does not impair osteoblast differentiation or mineralization, establishing its selectivity for osteoclasts—a critical safety consideration for anti-resorptive therapy. Collectively, while the previous study by our team reported that KM inhibits NF-κB activation via ubiquitination, the present study reveals that KM suppresses ERK and p38 phosphorylation in the MAPK pathway. The discrepancy regarding NF-κB warrants further investigation and may be attributable to differences in experimental conditions.

A particularly noteworthy finding of our study is the specificity of KM. At concentrations that effectively suppressed osteoclastogenesis, KM exhibited no cytotoxicity to BMMs (Fig. 1B)and, critically, did not impair the viability, differentiation, or mineralizing function of osteoblasts (Fig. 3). This selective targeting of osteoclasts is a highly desirable property for an anti-resorptive agent, as it minimizes the risk of disrupting bone formation, a common consideration in bone remodeling homeostasis^13,14,46^. The dynamic balance between osteoclasts and osteoblasts plays a crucial role in bone remodeling^46^, and our pit formation assays demonstrated that KM significantly inhibits the bone resorption function of osteoclasts without compromising osteoblast activity, suggesting a favorable safety profile for bone tissue.

The selective inhibition of osteoclasts by KM, without concomitant stimulation of osteoblast activity, distinguishes it from many naturally derived compounds previously reported to exert dual effects on bone remodeling. For instance, chitooligosaccharide has been shown to inhibit RANKL-induced osteoclastogenesis through suppression of MAPK/c-fos/NFATc1 signaling^23^, while melatonin inhibits osteoclast formation and osteolytic bone metastasis^15^. Similarly, isoalantolactone suppresses osteoclast differentiation via multiple signaling pathways^36^. In contrast, our data demonstrate that KM, at concentrations that effectively suppress osteoclast formation and function (10–40 µM), does not enhance osteoblast differentiation or mineralization (Fig. 3). This pharmacological profile positions KM as a selective anti-resorptive agent rather than a dual-action bone anabolic compound.

This apparent discrepancy—between KM’s lack of osteoblast stimulation and the osteogenic properties reported for many other natural products—warrants careful consideration. Several factors may account for this difference. First, the chemical structure and molecular targets of KM, an indole alkaloid, differ fundamentally from the polyphenolic compounds (e.g., flavonoids, stilbenes) commonly associated with osteogenic activity. While polyphenols often exert pleiotropic effects through antioxidant and estrogen receptor-mediated mechanisms, KM’s primary targets appear to be restricted to early signaling events in osteoclast precursors, specifically the MAPK pathway. Second, the concentration range used in our osteoblast experiments (up to 80 µM) was selected based on its efficacy in osteoclast assays; it remains possible that higher concentrations or different treatment durations might reveal osteogenic effects. However, such concentrations would exceed the non-cytotoxic threshold and lack translational relevance. Third, the cell context differs: osteoclasts and osteoblasts arise from distinct lineages (monocyte/macrophage vs. mesenchymal), and KM may selectively interact with signaling components uniquely expressed or regulated in osteoclast precursors.

This selectivity, rather than being a limitation, represents a distinctive pharmacological advantage. Dual-action agents that both inhibit osteoclasts and stimulate osteoblasts, while theoretically attractive, carry inherent risks of disrupting the finely tuned coupling between bone resorption and formation. Excessive osteoblast stimulation in the context of suppressed osteoclast activity could lead to aberrant bone remodeling or off-target effects. In contrast, selective anti-resorptive agents such as KM, which do not interfere with osteoblast function, may offer a safer profile for long-term management of osteolytic diseases, particularly in settings where bone formation is already compromised, such as aging or glucocorticoid-induced osteoporosis. Moreover, the specificity of KM for osteoclasts, combined with its inhibition of the MAPK pathway, provides a mechanistically distinct entry point for therapeutic intervention, complementing existing agents that target osteoclast differentiation or survival through other mechanisms (e.g., bisphosphonates, denosumab).

The translational relevance of our in vitro findings was strongly confirmed in vivo. In the OVX mouse model, a well-established model for postmenopausal osteoporosis^36–38^, KM administration significantly preserved bone mass and improved trabecular microarchitecture parameters, including BV/TV and Tb.N (Fig. 4A, E). Most importantly, the reduction in the number of TRAcP-positive osteoclasts on the bone surface of KM-treated mice(Fig. 4C, D) directly mirrors our in vitro observations, providing compelling evidence that the bone-protective effect of KM is primarily mediated through the suppression of osteoclast activity. The inhibition of the MAPK pathway by KM, together with its lack of effect on NF-κB, provides strategic insight into its mechanism of action. Current research indicates that nearly every extracellular ligand involved in skeletal biology exerts its effects, at least in part, through the MAPK pathway^44^, and the NF-κB pathway is equally critical^23^. Given that these pathways can exhibit compensatory cross-talk^42^, simultaneously targeting both may yield a more robust and effective suppression of osteoclastogenesis compared to single-pathway inhibitors, as suggested by other studies^23^. This multi-target characteristic, coupled with its specificity for osteoclasts, underscores the unique value of KM.

### Limitations

Several limitations should be acknowledged. First, consistent with our previous study (You et al., 2024^47^), in which KM at 5 mg/kg showed no hepatotoxicity or nephrotoxicity as evidenced by normal serum ALT, AST, BUN, and creatinine levels as well as unremarkable liver and kidney histology (Figure. 5E, F in that study), no mortality, abnormal behavior, or gross organ abnormalities were observed at the administered doses (1 and 10 mg/kg) in the present study. Although comprehensive pharmacokinetic and toxicological evaluations (e.g., LD50) were not performed, the available evidence supports a favorable safety profile of KM at the doses used. The direct molecular target of KM also remains to be determined. Second, the OVX mouse model, although well-established, does not fully recapitulate the multifactorial nature of human osteoporosis. Third, the concentration of KM used in Western blotting (20 µM) was lower than that in functional assays (40 µM) to minimize non-specific cellular stress. Future studies should focus on target identification, safety profiling, and validation in additional osteolytic models.

In conclusion, our study identifies KM as a novel and specific inhibitor of osteoclast formation and function. Its mechanism of action involves the suppression of the RANKL-induced MAPK signaling pathway, while NF-κB does not appear to be a primary target of KM under the conditions tested. These findings not only shed light on the pharmacological basis of KM’s effects on bone but also pave the way for its future development as a potential therapeutic agent for osteolytic diseases such as osteoporosis and cancer bone metastasis.

## Conclusion

In summary, our study has elucidated that the inhibitory effects of KM on osteoclasts are primarily manifested through the suppression of their differentiation and functional activation. Mechanistically, KM inhibits RANKL-induced phosphorylation of ERK and p38 in the MAPK pathway, while having no significant effect on NF-κB p65 phosphorylation. Our findings confirm that KM effectively inhibits osteoclastogenesis and lytic bone destruction through MAPK signaling, supporting its potential as a therapeutic agent for osteolytic bone diseases. These findings support the potential of KM as a therapeutic agent for the prevention and treatment of osteolytic bone diseases. Future research should focus on validating these findings through clinical trials and developing KM as a novel therapeutic option for patients suffering from lytic bone destruction.

## Supplementary Information

Below is the link to the electronic supplementary material.


Supplementary Material 1


## Data Availability

The datasets generated during and/or analysed during the current study are available from the corresponding author on reasonable request.

## Abbreviations

KM: Koumine
α-MEM: MEM with alpha modification
BMMs: Bone marrow-derived macrophages
BV: Bone volume
BV/TV: Bone volume/total volume
CCK-8: Cell counting kit-8
CTSK: Cathepsin K
DMSO: Dimethyl sulfoxide
DMEM: Dulbecco’s modified eagle medium
FBS: Fetal bovine serum
H&E: Hematoxylin and eosin
M-CSF: Macrophage colony-stimulating factor
Micro-CT: Micro-computed tomography
NFATc1: Nuclear factor of activated T-cells 1
NF-κB: Nuclear factor-κB
OD: Optical density
PBS: Phosphate-buffered saline
PS: Penicillin/streptomycin
RANKL: Receptor activator of nuclear factor-κB ligand
TRAcP: Tartrate-resistant acid phosphatase
TV: Tissue volume
Tb.N: Trabecular number
Tb.Th: Trabecular thickness
Tb.Sp: Trabecular separation
MAPK: Mitogen-activated protein kinase
