# Supplementary material for: Koumine inhibits osteoclastogenesis and prevents ovariectomy-induced bone loss via suppression of the MAPK signaling pathways
Source: Sci Rep. 2026 Jun 13;16:27051. doi: 10.1038/s41598-026-57325-4 (PMC13522517; doi:10.1038/s41598-026-57325-4)

p-ERK

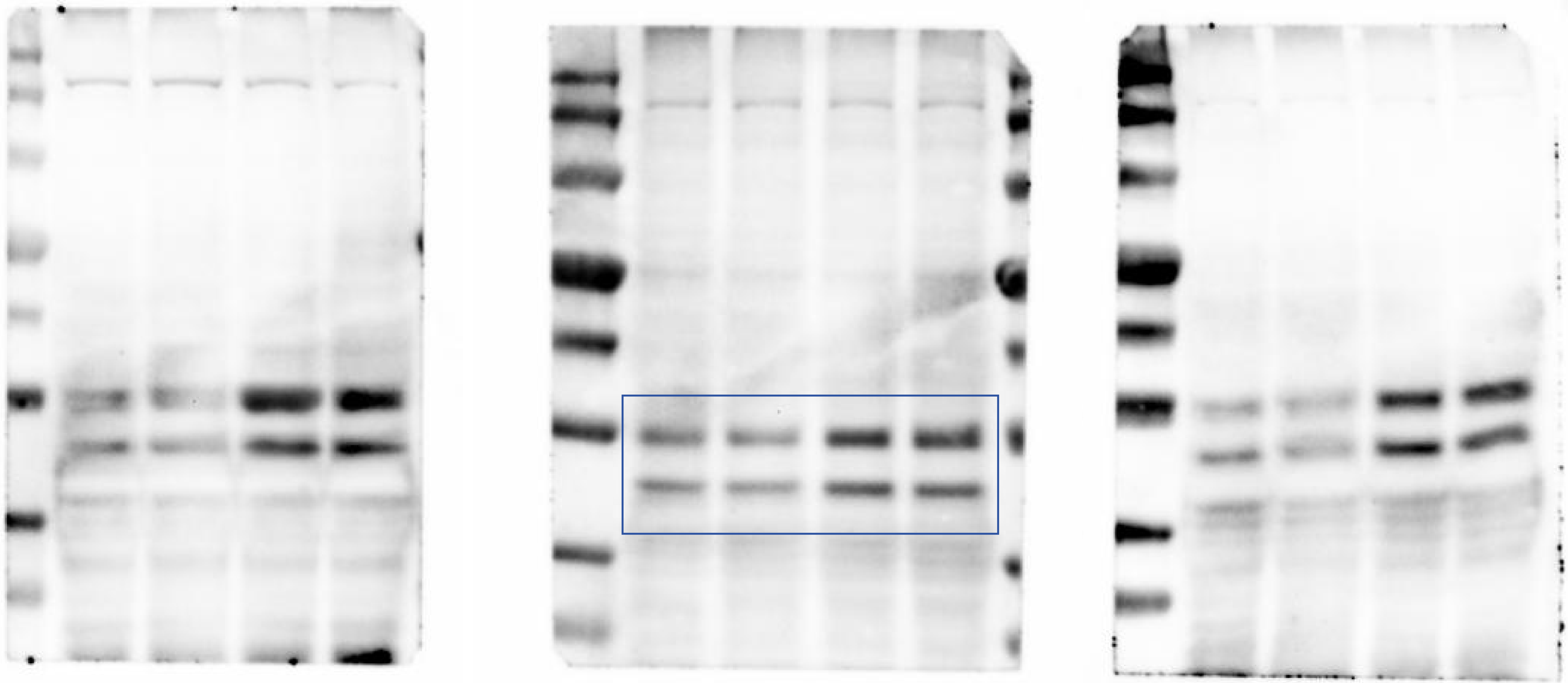

ERK

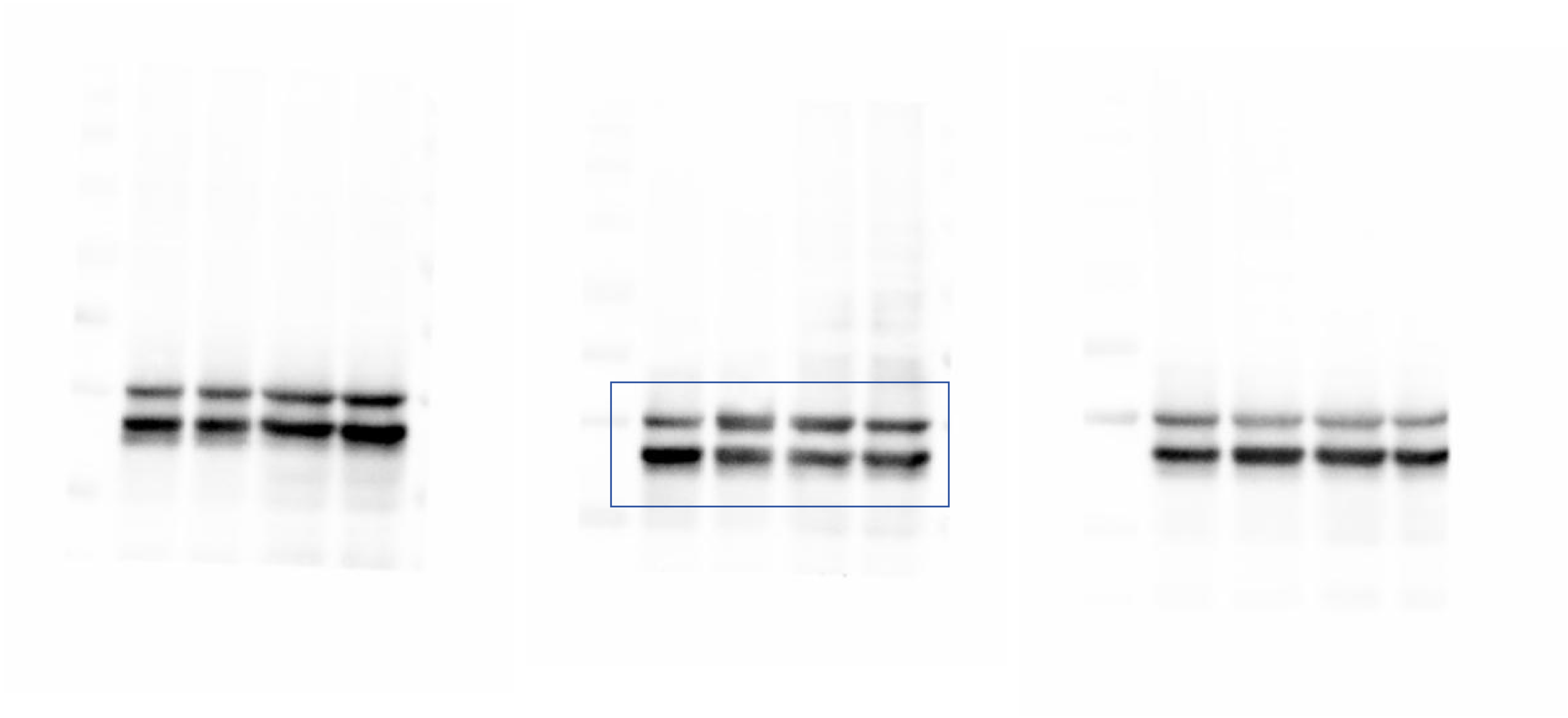

p-JNK

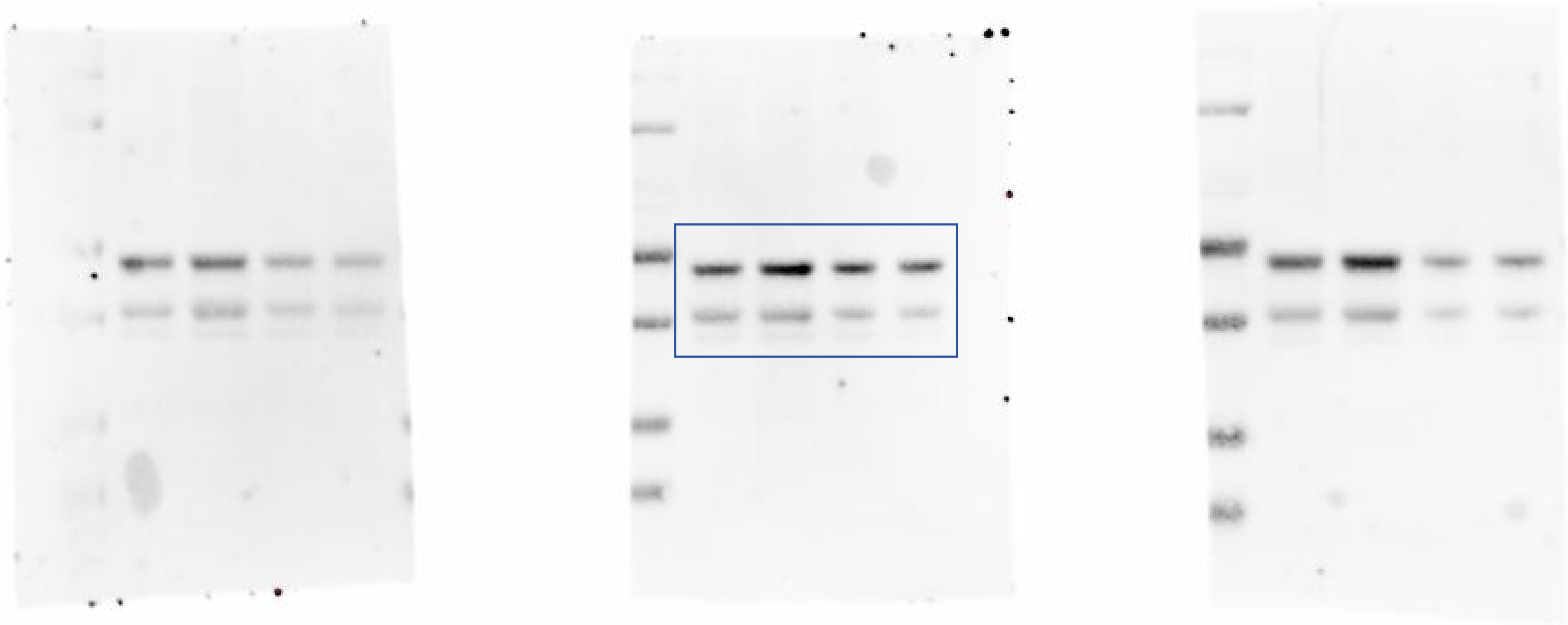

JNK

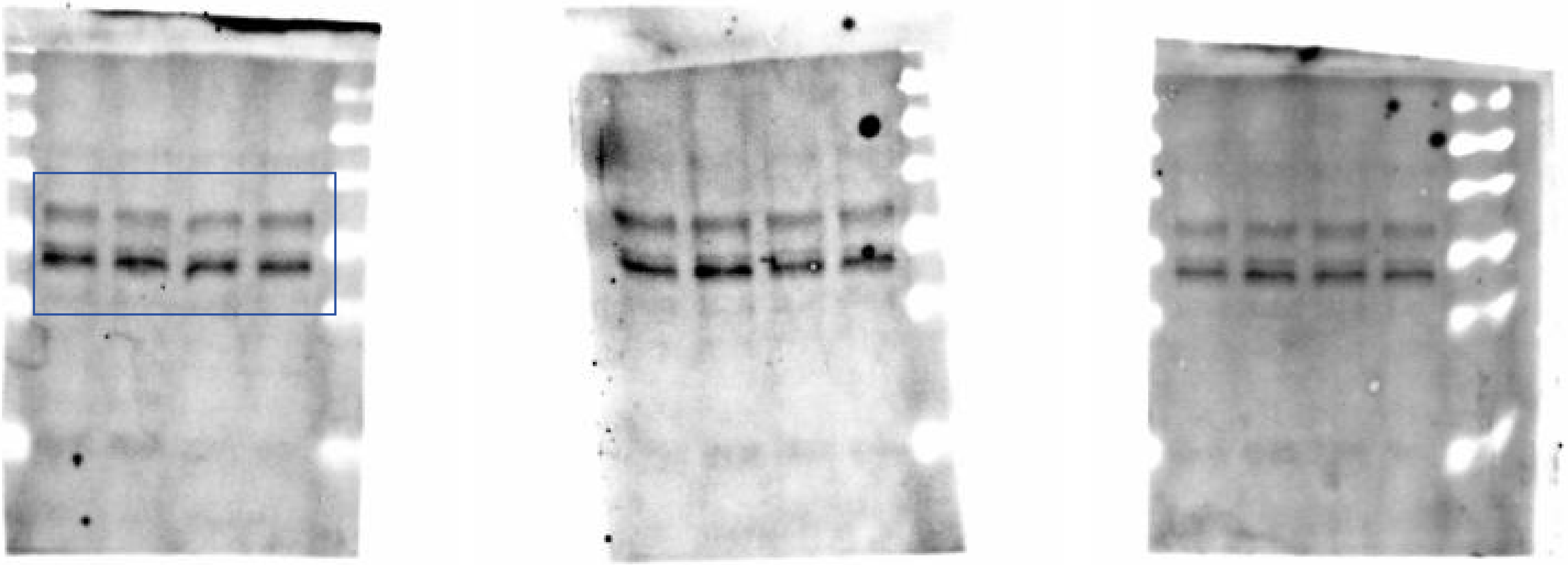

p-p38

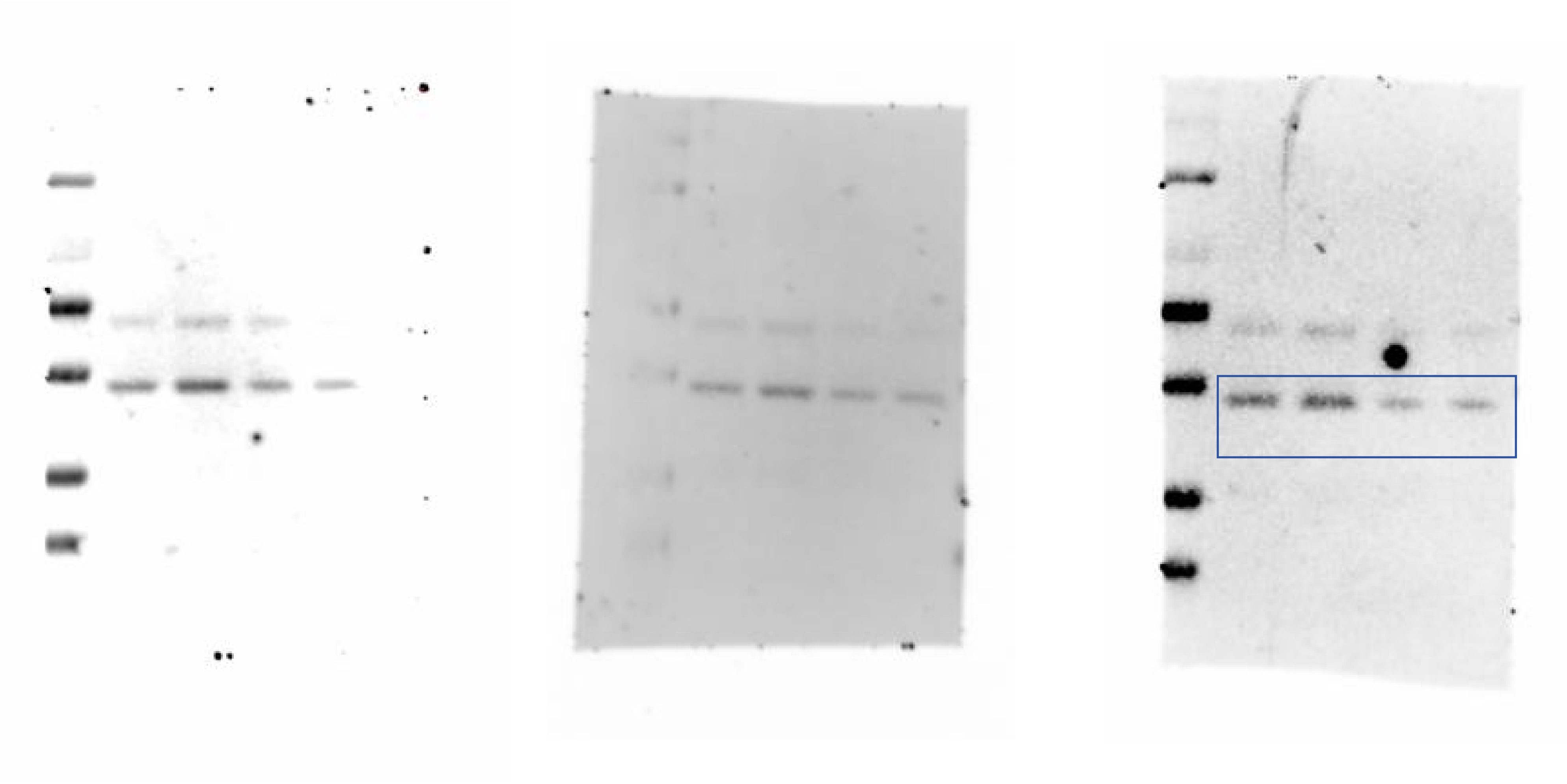

p38

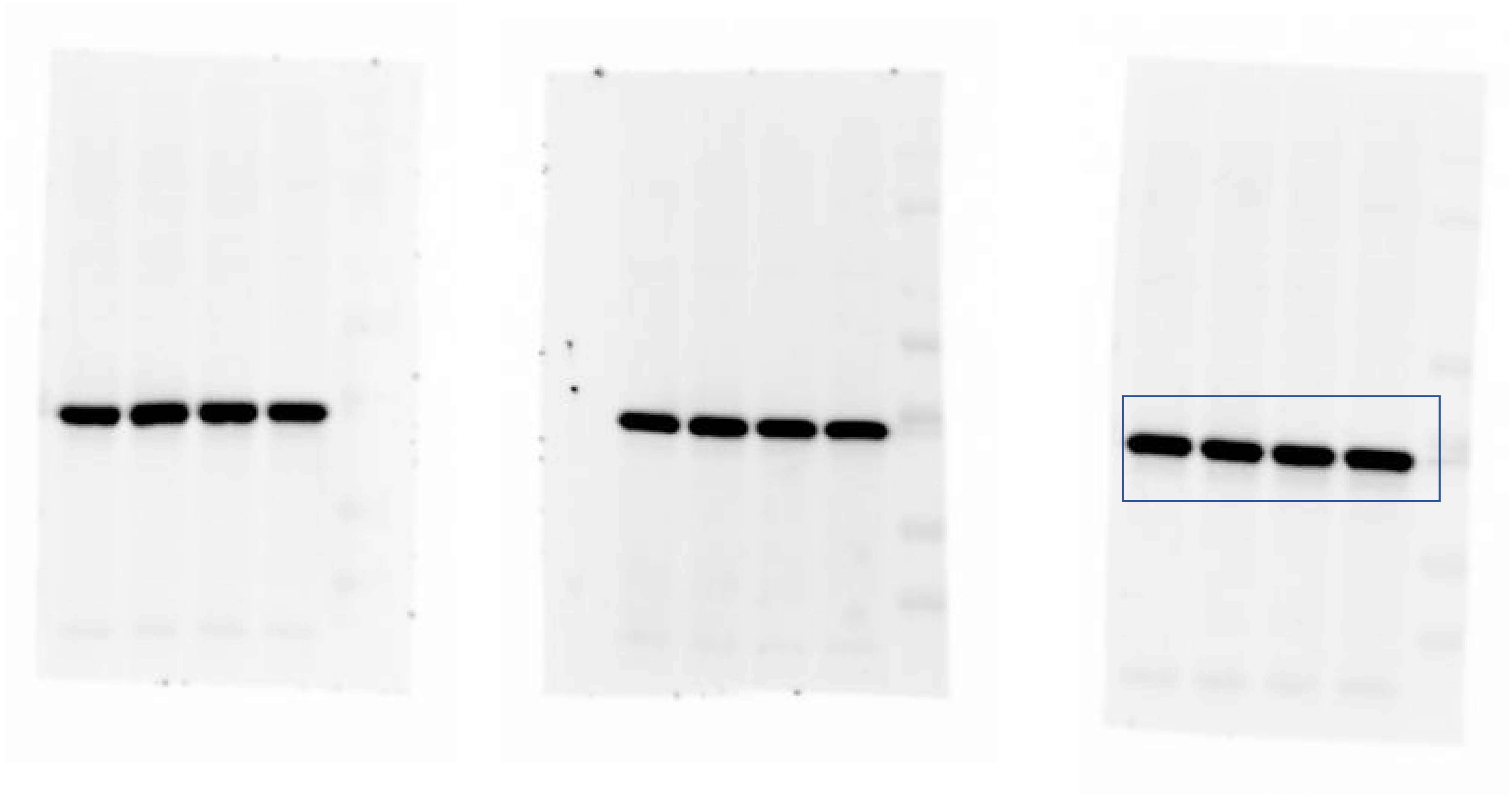

p-p65

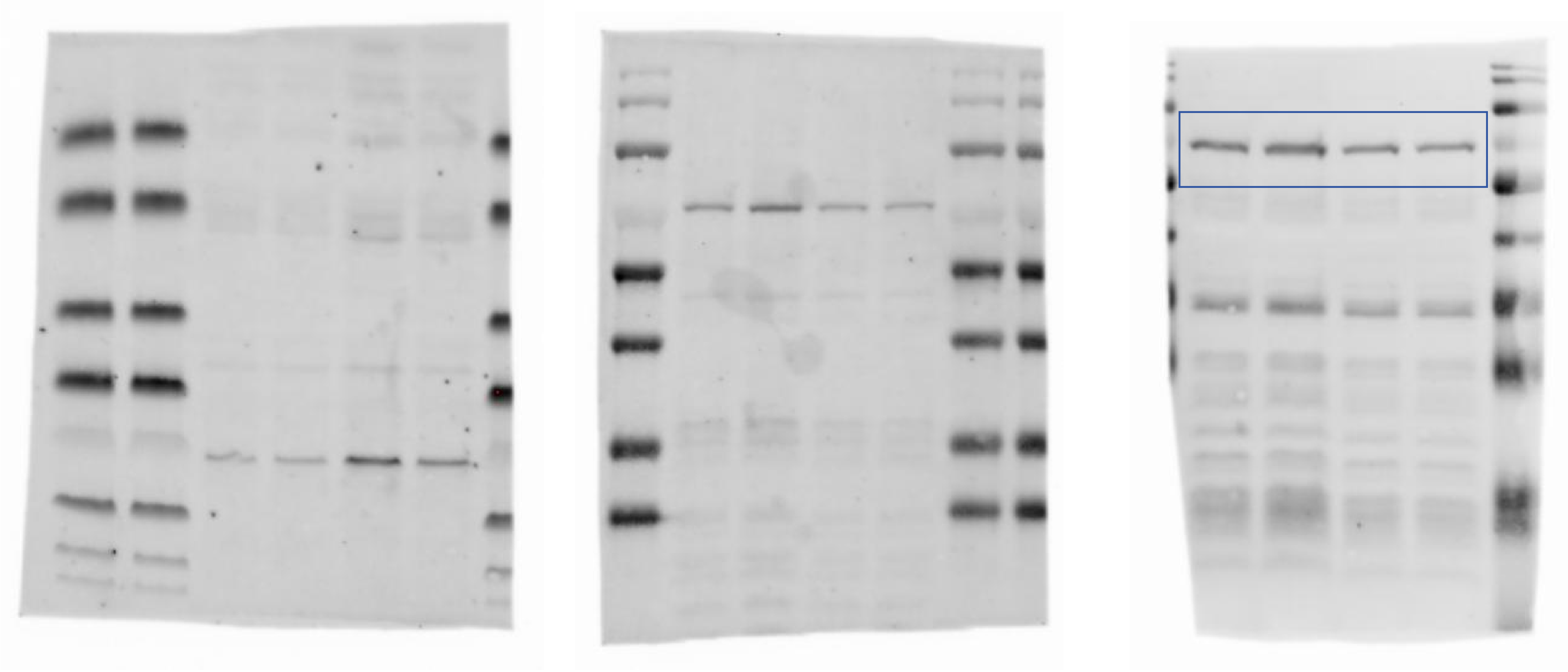

p65

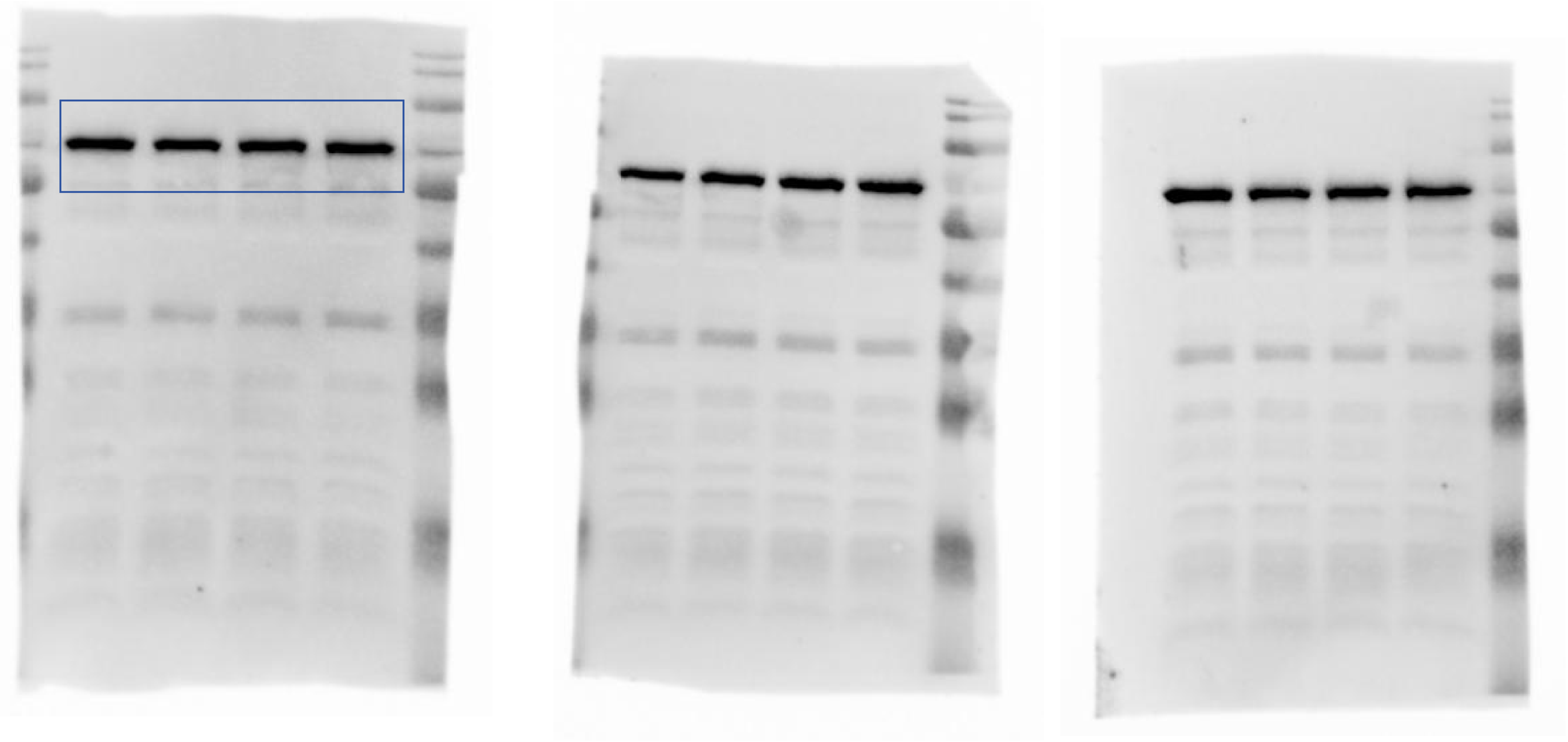

GADAP

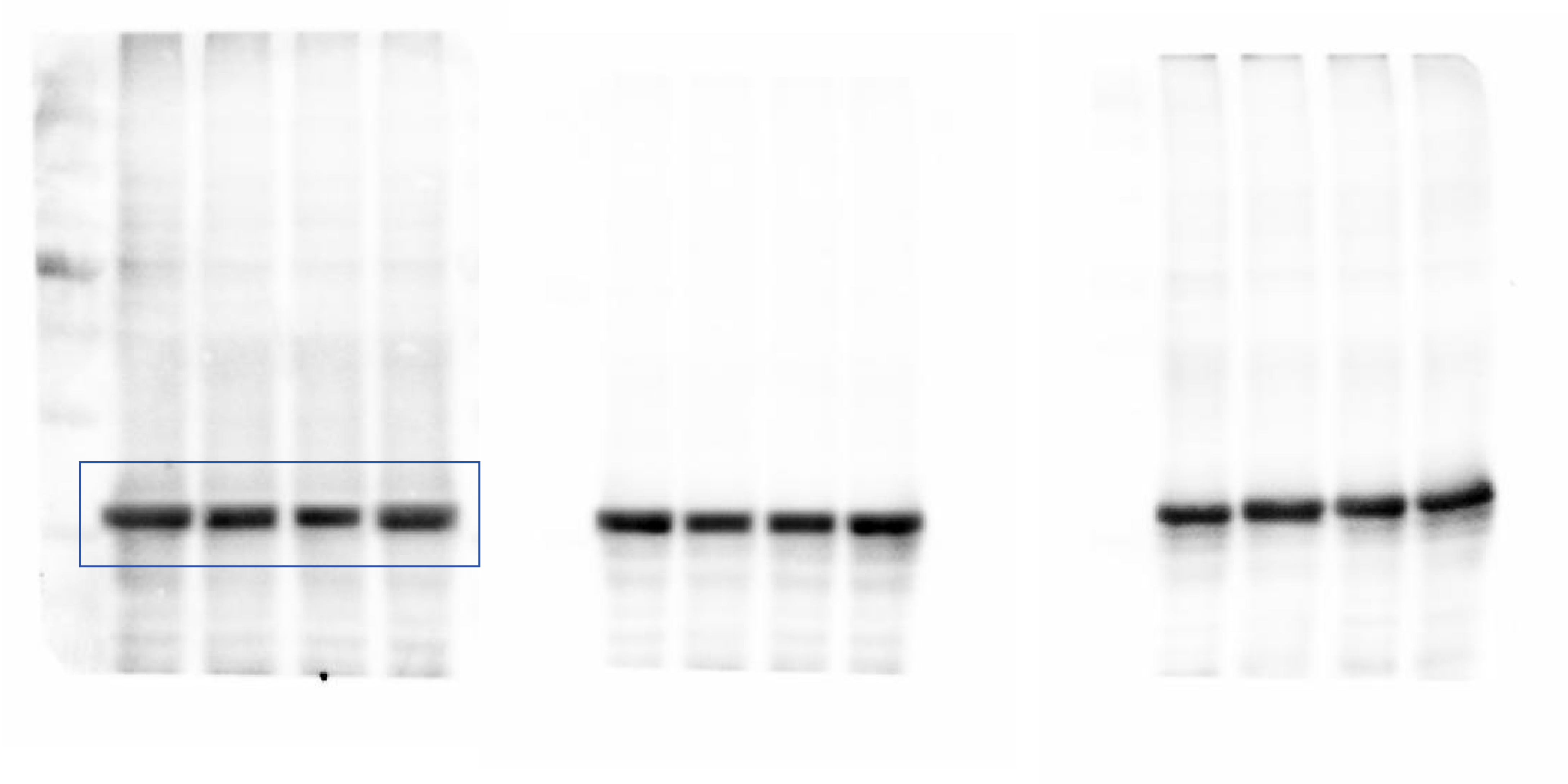

Supplement: Supplementary file 1 [file 41598_2026_57325_MOESM1_ESM.pdf]
